# Mechanistic Insights into the Role of OPN in Mediating Brain Damage via Triggering Lysosomal Damage in Microglia/Macrophage

**DOI:** 10.3390/cells12060854

**Published:** 2023-03-09

**Authors:** Chengcheng Gai, Yijing Zhao, Danqing Xin, Tingting Li, Yahong Cheng, Zige Jiang, Yan Song, Dexiang Liu, Zhen Wang

**Affiliations:** 1 Department of Physiology, School of Basic Medical Sciences, Cheeloo College of Medicine, Shandong University, Jinan 250012, China; 2 Department of Medical Psychology and Ethics, School of Basic Medical Sciences, Cheeloo College of Medicine, Shandong University, Jinan 250012, China

**Keywords:** osteopontin, galectin-3, microglia/macrophages, lysosomal, cathepsin B, hypoxia–ischemia

## Abstract

We previously found that osteopontin (OPN) played a role in hypoxia–ischemia (HI) brain damage. However, its underlying mechanism is still unknown. Bioinformatics analysis revealed that the OPN protein was linked to the lysosomal cathepsin B (CTSB) and galectin-3 (GAL-3) proteins after HI exposure. In the present study, we tested the hypothesis that OPN was able to play a critical role in the lysosomal damage of microglia/macrophages following HI insult in neonatal mice. The results showed that OPN expression was enhanced, especially in microglia/macrophages, and colocalized with lysosomal-associated membrane protein 1 (LAMP1) and GAL-3; this was accompanied by increased LAMP1 and GAL-3 expression, CTSB leakage, as well as impairment of autophagic flux in the early stage of the HI process. In addition, the knockdown of OPN expression markedly restored lysosomal function with significant improvements in the autophagic flux after HI insult. Interestingly, cleavage of OPN was observed in the ipsilateral cortex following HI. The wild-type OPN and C-terminal OPN (Leu152-Asn294), rather than N-terminal OPN (Met1-Gly151), interacted with GAL-3 to induce lysosomal damage. Furthermore, the secreted OPN stimulated lysosomal damage by binding to CD44 in microglia in vitro. Collectively, this study demonstrated that upregulated OPN in microglia/macrophages and its cleavage product was able to interact with GAL-3, and secreted OPN combined with CD44, leading to lysosomal damage and exacerbating autophagosome accumulation after HI exposure.

## 1. Introduction

Osteopontin (OPN), also called secreted phosphoprotein 1, is an extracellular matrix protein involved in many physiological and pathophysiological processes [1]. The basal OPN level in adult brains is weak [2], while OPN expression is notably upregulated during the inflammation associated with Alzheimer’s disease and other neurodegenerative conditions [3,4]. Several studies have suggested the immuno-modulatory role of OPN in brain pathologies [5,6]. Our previous studies have shown that hypoxia–ischemia (HI) brain damage increased OPN expression in microglia/macrophages of neonatal mice, which contributed to neuroinflammation [7,8].

Autophagy is an evolutionarily conserved transport pathway crucial to maintaining cellular homeostasis through the sequestration, delivery, and degradation of unwanted proteins, macromolecular complexes, and organelles into lysosomes. Lysosomes are cytoplasmic membrane-enclosed organelles containing hydrolytic enzymes that participate in the control of the intracellular turnover of macromolecules [9]. A study has shown that lysosomal destabilization by oxidative stress and other apoptotic signals causes the leakage of cathepsins and other hydrolases into the cytosol, which is potentially harmful to cell survival [10]. For example, the leakage of cathepsin B (CTSB) from the lysosomes to the cytoplasm could trigger the activation of the NOD-like receptor thermal protein domain-associated protein 3 (NLRP3) inflammasome in microglia and additionally contribute to NLRP3-mediated pyroptosis [11,12]. Given the essential role of lysosomes in the maturation/degradation stage of autophagy, it is possible that progressive dysfunction in the lysosomal apparatus is deleterious to cells.

Based on our previous studies, the aim of this study was to evaluate the effect of OPN on impairing the lysosomal function and autophagic flux following HI insult. We found that intracellular OPN interacted with galectin-3 (GAL-3) to induce lysosomal damage and further identified the binding region of OPN to GAL-3 as the C-terminal fragment. It was also found that the secreted OPN regulated lysosomal function by binding to its receptor CD44. The study provides novel mechanistic insights into the pathophysiology of lysosomal dysfunction after HI exposure.

## 2. Methods

### 2.1. Ethical Statement

Ethics approval statements for animal work and procedures were provided by the Animal Ethical and Welfare Committee of Shandong University (approval No. ECSBMSSDU2020-2-067).

### 2.2. Animals and Treatment

C57BL/6J mice (female, 3–5 g) provided by the Laboratory Animal Center of Shandong University (Jinan, China) were used in all experiments. A well-characterized model of neonatal HI was followed as previously described [13]. C57BL/6 mouse pups were anesthetized with 2% isoflurane on postnatal day 7 (P7) and the right pulsating common carotid artery was carefully separated and double-ligated. The skin incision was sutured with a surgical suture. After surgery, the pups were returned to their dam for 30 min. The pups were then placed in a hypoxic chamber for 1 h. The chamber was maintained at 37 °C and contained 9.5% O_2_ + 90.5% N_2_.

Mice in HI+3-methyladenine (3-MA, Cat# M9281, Sigma-Aldrich, St. Louis, MO, USA) and HI+chloroquine (CQ, Cat# C6628, Sigma-Aldrich, St. Louis, MO, USA) groups were administered 3-MA (10 mg/kg) or CQ (10 mg/kg) intraperitoneally at 1 h, 1 day, 2 days, and 3 days after HI insult [14,15,16], respectively. Finally, the mice were sacrificed to obtain the injured cortex for experimentation.

### 2.3. Sample Preparation for Tandem Mass Tag (TMT) Analysis

This study randomly selected three right cerebral cortices from the Sham and HI samples, respectively, for TMT-based quantitative proteomics analysis. The samples were sent to Novogene Bioinformatics Technology Co. (Beijing, China) for TMT quantitative proteomics using a C18 column (Waters BEH C18 4.6 mm × 250 mm, 5 µm) on a Rigol L3000 HPLC. Statistical analysis for the proteomic data was performed using Proteome Discoverer software (2.2; Thermo Fisher Scientific, Waltham, MA, USA).

### 2.4. Protein–Protein Interaction (PPI) Network

The PPI network was analyzed with the Cytoscape software (3.7, San Diego, CA, USA) by submitting sequences of differentially expressed proteins (DEPs), and an interaction with a combined score > 0.4 was considered as statistically significant.

### 2.5. Kyoto Encyclopedia of Genes and Genomes (KEGG) Analyses

To identify the biological functions of the selected DEPs in the Sham and HI groups, the determined protein sequences were mapped using KEGG pathway enrichment analyses.

### 2.6. Lentiviral and Plasmid Transfection

Lentiviral OPN, GAL-3 shRNA (si-OPN, si-GAL-3), lentiviral shRNA negative control (si-NC), and LC3-GFP plasmid were packaged in Genechem (Shanghai, China), and mouse CD44 small interfering RNA (si-CD44) and the negative control (si-NC) were obtained from Genechem (Shanghai, China). The GAL-3-mCherry, OPN-EGFP, wild-type OPN-Flag (OPN-WT-Flag), the N-terminal fragment of OPN (Met1-Gly151, OPN-N-Flag), and C-terminal fragment of OPN (Leu152-Asn294, OPN-C-Flag) plasmids were purchased from Miaoling Plasmidbio (Wuhan, China). Transfection of the lentivirus, plasmid, and RNA interference into cells was carried out using Lipofectamine 2000 transfection reagent (Cat# 11668019, Invitrogen, Carlsbad, CA, USA) following the manufacturer’s protocol.

### 2.7. Oxygen–Glucose Deprivation (OGD)

BV-2 cells were obtained from Cellcook (Guangzhou Cellcook Biotech Co., Ltd., Guangzhou, China). BV-2 was used to imitate primary microglia for the observation of pathophysiological processes in microglia [17]. BV-2 cells seeded in 6-well plates were incubated overnight and treated with OGD. The culture medium was replaced with glucose-free Dulbecco’s modified eagle medium, and the culture dish was placed in a hypoxia incubator with a 1% O_2_ and 5% CO_2_ mixture for 3 h. After OGD, the cells were incubated for 24 h in normal oxygen. The cells were then collected at the time points indicated for use in future experiments.

### 2.8. Lyso-Tracker Red and Mito-Tracker Red CMXRos Staining

Lyso-Tracker Red (Cat# C1046, Beyotime, Shanghai, China) staining was used to assess lysosomal activity. Lyso-Tracker Red was dissolved in phosphate-buffered saline (PBS) and stored at 4 °C. Prior to imaging, Lyso-Tracker Red (50 mol/L) was preheated at 37 °C and incubated with BV-2 cells or HEK293T cells for 30 min at 37 °C. Cell nuclei were stained with 4′,6-diamidino-2-phenylindole (DAPI) at room temperature for 10 min. Images were taken on a fluorescence microscope (Axio Vert.A1, Carl Zeiss, Oberkochen, Baden-Württemberg, Germany).

Mito-Tracker Red CMXRos purchased from Beyotime (Cat# C1035). Mito-Tracker Red CMXRos (50 mol/L) was incubated with HEK293T cells for 30 min at 37 °C. The cell nuclei were stained with DAPI at room temperature for 10 min. Images were taken on a Carl Zeiss (Axio Vert.A1) fluorescence microscope. Data analysis was performed using ImageJ software.

### 2.9. Analysis of Lysosome Membrane Permeability

Acridine orange (AO) (Cat# YS175111, Solarbio, Beijing, China) staining was used to analyze the lysosomal membrane permeability as previously described [18]. AO is a lysosomotropic metachromatic fluorochrome and emits red fluorescence in intact lysosomes and green fluorescence in the cytoplasm [10]. BV-2 cells were cultured in 24-well chamber slides and treated without or with OGD. The cells were then incubated with 1 μg/mL AO for 30 min at 37 °C in the dark. The cells were visualized with a Carl Zeiss (Axio Vert.A1) fluorescence microscope. Data analysis was performed using ImageJ software.

### 2.10. Infarct Volume Measurement

At 3 days post-HI, the brains were obtained and sectioned into 4 slices, and then immersed in 2% 2, 3, 5-triphenyltetrazolium chloride monohydrate (TTC, Cat# T8877, Sigma-Aldrich, St. Louis, MO, USA) solution at 37 °C for 20 min. The infarct volume was traced and analyzed using ImageJ software (1.53c, NIH, Bethesda, MD, USA). Infract volume quantification was then conducted following procedures described previously [19].

### 2.11. Lateral Cerebral Ventricle Injections

P4 mouse pups were fixed in a prone position under isoflurane anesthesia. The injection site was positioned at the midpoint of the lambda and bregma sutures, 0.8–1 mm laterally from the sagittal suture [20]. Then, 3 × 10^7^ TU/3 μL of si-OPN, si-GAL-3, and si-NC; 3 μL si-CD44 or si-NC at a concentration of 20 nmol/L were injected into the lateral ventricle (3 mm deep from the skull). The injection rate was 0.5 μL/min and the needle remained at the site for about 5 min after injection. The injection needle was then raised carefully at a speed of 1 mm per minute.

### 2.12. Immunofluorescence Staining

Four-micrometer paraffin sections were baked, deparaffinized, and rehydrated in xylene and graded alcohols; antigen retrieval was then performed using citrate buffer (Cat# C1032, Solarbio, Beijing, China) for 20 min at 100 °C within a pressure chamber. Slides were blocked using normal 10% goat serum or donkey serum for 1 h at room temperature, and subsequently incubated with indicated primary antibodies (Dilution: 1:200) at 4 °C overnight. Sections were then incubated for 1 h at 37 °C with the fluorescein-conjugated secondary antibodies, and the sections were then counterstained with DAPI. The negative control consisted of sections processed in the same way as the tests with the omission of the primary antibody incubation step. Images were examined through Carl Zeiss (Axio Vert.A1) fluorescence microscopy. All experiments were performed three times. There were four mice in each experimental group, and two sections per mouse were randomly selected for imaging. Three fields of the image were randomly selected for each section for counting. Counting was performed in a blinded manner. Data analysis was performed using ImageJ software. All antibodies used in the study are shown in Table 1.

### 2.13. Terminal Deoxynucleotidyltransferase-Mediated dUTP-Biotin Nick End Labeling (TUNEL) Assay

The FITC-labeled TUNEL apoptosis assay (Cat# G1501, Servicebio, Wuhan, China) was performed to detect apoptosis in frozen slides of brain tissue following the manufacturer’s protocol. The frozen slides were restored to room temperature and dried in air, and then incubated for 20 min at 37 °C with a proteinase K working solution. The TUNEL reaction mixture (recombinant TdT enzyme: FITC-12-dUTP; labeling mix: equilibration buffer = 1 µL:5 µL:50 µL) was added to the samples. The samples were capped and incubated for 60 min at 37 °C in a humidified atmosphere in the dark. The slides were rinsed 3 times with PBS and counterstained with DAPI staining for 10 min. For the negative controls, the TdT enzyme was not added to the samples. Immunofluorescence images were obtained using a fluorescence microscope (Olympus BX53, Tokyo, Japan). There were four mice in each experimental group, and two sections per mouse were randomly selected for imaging. Three fields of the image were randomly selected for each section for counting. Counting was performed in a blinded manner. Data analysis was performed using ImageJ software.

### 2.14. Co-Immunoprecipitation (IP) Assay

For co-IP in cells, the whole-cell lysates were incubated with protein A + G agarose beads conjugated with antibodies against OPN with rocking at 4 °C overnight. Immunoglobulin G (IgG) from the same animal species was used as the negative control. The beads were eluted five times with IP buffer and neutralized with 40 μL of 5 × SDS-PAGE loading buffer to heat at 100 °C for 10 min. IP and input samples were subjected to Western blot analyses.

For the in vitro co-IP assay, OPN and GAL-3 proteins were generated in vitro through translation using TnT^®^ T7 Coupled Reticulocyte Lysate Systems (Cat# L4600, Madison, Promega, WI, USA) according to the manufacturer’s protocol. The same analysis as that described above was performed after the protein products were combined.

### 2.15. Neurobehavioral Evaluation

#### 2.15.1. Geotaxis Reflex

Mice were placed with their heads facing downward on the center of a 30 cm grid inclined at an angle of 45 The time required for a full angle of 90 upward rotation within a maximum time of 30 s was recorded. Each mouse was tested 4 times and the average value from the 4 tests was calculated.

#### 2.15.2. Grip Test

The forelimbs of pups were placed on a 30 cm thin iron wire to assess their grip strength. The latent time to fall was recorded with a maximum time allowance of 60 s. Each mouse was tested 4 times and the average value from the 4 tests was calculated.

#### 2.15.3. T-Maze

T-maze alternation was used to determine cognitive impairment. For the first forced trial, the mouse was placed in the start arm; once it entered the goal arm, the goal arm was immediately blocked with a plastic guillotine door. The mouse was blocked in the chosen arm for 30 s. The mouse eventually returned to the start arm and the next trial was started. In the second run, with all of the arms unblocked, the mouse had to choose between the previously entered goal arm (no alternation) and a new arm (alternation) [21]. The test procedures were repeated 4 times per day for 3 consecutive days.

$$\mathrm{Alternation}\;\left(\%\right)=\frac{\mathrm{The}\;\mathrm{number}\;\mathrm{of}\;\mathrm{correct}\;\mathrm{choices}}{\mathrm{Total}\;\mathrm{sets}\;\mathrm{performed}}\times 100\%$$

$$\mathrm{Side}\;\mathrm{preference}\;\left(\%\right)=\frac{\mathrm{The}\;\mathrm{number}\;\mathrm{of}\;\mathrm{preferred}\;\mathrm{side}\;\mathrm{that}\;\mathrm{the}\;\mathrm{mouse}\;\mathrm{has}\;\mathrm{chosen}}{\mathrm{Total}\;\mathrm{runs}\;\mathrm{performed}}\times 100\%$$

### 2.16. Quantitative Real-Time Polymerase Chain Reaction (qRT-PCR)

The total RNA of tissue samples was extracted using an Ultrapure RNA Kit (Cat# CW0581M, CWBIO, Beijing, China) following the manufacturer’s protocol. The purity of the RNA was measured using a spectrophotometer at A260 nm and A280 nm, and samples with an A260/A280 ratio of ≥1.8 were used. A Revert Aid First Strand cDNA Synthesis Kit (Cat# FSQ-101, TOYOBO, Osaka, Japan) was used to synthesize first-strand cDNA with 1 μg of total RNA and primers. The qRT-PCR was completed with a SYBR Green PCR master mix (Cat# PC3301, Aidlab Biotechnologies, Beijing, China) using a Bio-Rad IQ5 Real-Time PCR System (Bio-Rad, Hercules, CA, USA). The reaction annealing temperature was 60 °C and amplification was performed for 40 cycles. The reverse transcription negative control (without reverse transcriptase) was included to confirm the absence of genomic DNA in the reactions in qRT-PCR analysis. The primer sequences used are described in Table 2.

### 2.17. Western Blot Analysis

The ipsilateral cortexes from mice (*n*  =  4 in each group) were lysed in RIPA buffer (Cat# P0013B, Beyotime) containing protease inhibitors and phosphatase inhibitor. A lysosome isolation kit (Cat# Lysiso1, Sigma-Aldrich, St. Louis, MO, USA) was used to obtain the lysosomes and the remaining cytoplasmic components in the brain tissue, and the cytoplasmic components were added to RIPA buffer for lysing to obtain cytoplasmic protein. The protein concentrations were determined using a BCA Protein Assay Kit (Cat# CW0014, CWBIO, Beijing, China). The protein sample lysates were electrophoretically separated on an SDS-PAGE (10% or 12%), then transferred from gels to 0.45 µm polyvinylidene fluoride membranes (Cat# IPVH00010, Millipore, Boston, MA, USA). The membranes with proteins were blocked for 1 h with 5% non-fat milk in TBST at room temperature and then incubated overnight at 4 °C with the following primary antibodies (Dilution: 1:1000). An enhanced chemiluminescence (Cat# WBKLS0500, Millipore, Boston, MA, USA) system was used to monitor the blot signals. All experiments were performed three times. Protein quantitative analysis was conducted using ImageJ software. All antibodies used in the method are shown in Table 1.

### 2.18. Statistical Analysis

The data from all experiments were analyzed using SPSS software. All of the experiments were carried out in a blinded manner. The data were presented as the mean ± SD of 4 or more biologically independent replicates. All experiments were performed three times. Normality between group samples was assessed using the Shapiro–Wilk test. The statistical significance of two independent groups was evaluated using Student’s *t*-test. One-way analysis of variance (ANOVA) followed by the Dunnett corrections or the Bonferroni corrections were used for comparisons of three or more groups. Statistical significance was defined as * *p* < 0.05; ** *p* < 0.01; *** *p* < 0.001.

## 3. Results

### 3.1. Up-Regulated OPN Colocalized with LAMP1 and GAL-3 in Microglia/Macrophages after HI Insult

**Figure 1:**
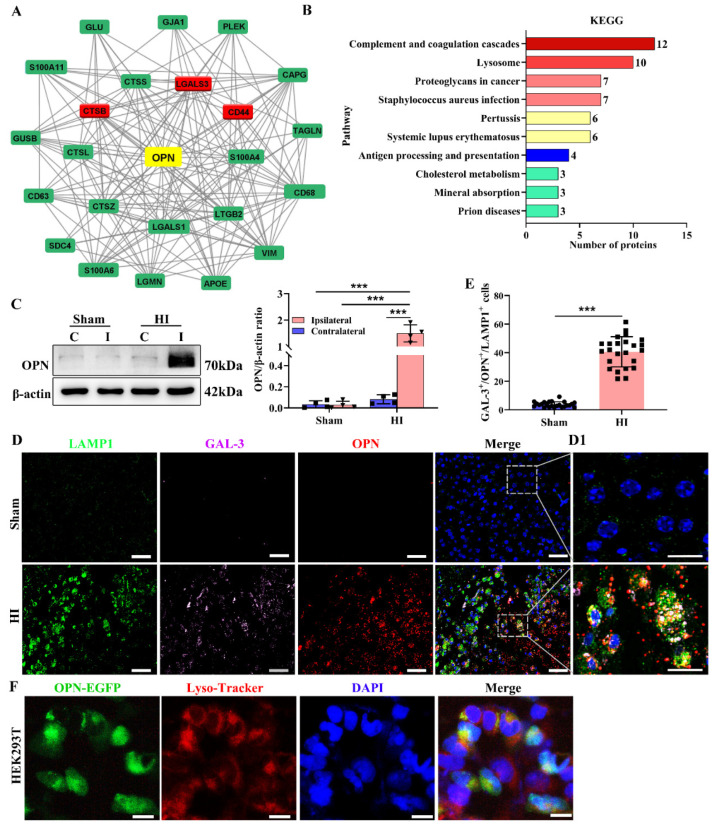
Increased expression of OPN was colocalized with the lysosome. (**A**) Interaction analysis of OPN-related target proteins. (**B**) Bar diagram of KEGG pathways from the comparison between the Sham group and HI group. (**C**) Western blot demonstrated upregulated OPN in ipsilateral regions (abbreviated as I) in the matching contralateral (abbreviated as C) region of HI and Sham group (*n* = 4). All experiments were performed three times. (**D**,**E**) Immunofluorescence images revealing colocalization of LAMP1, GAL-3, and OPN at 3 days post-HI (*n* = 4), Scale bar = 50 μm; D1: Magnified views of boxed regions in (**D**) showing colocalization of LAMP1, GAL-3, and OPN. Scale bar = 20 μm. (**F**) HEK293T cells were stably transfected with OPN-EGFP and labeled with Lyso-Tracker Red (*n* = 6). Scale bar = 20 μm. Values represent the mean ± SD, *** *p* < 0.001 according to one-way ANOVA with Bonferroni corrections in (**C**). *** *p* < 0.001 according to *t*-test in (**E**), contralateral; GAL-3, galectin-3; HI, hypoxia-ischemia; I, ipsilateral; KEGG, Kyoto encyclopedia of genes and genomes; OPN, osteopontin.

We previously found that OPN protein expression was largely increased in the ipsilateral cortex and located in the microglia post-HI [8]. To explore the function of OPN, the Cytoscape software was used to predict OPN-interacting proteins and the KEGG pathway. The TMT-based quantitative proteomic analysis and PPI network of OPN revealed that a total of 23 DEPs exhibited a potential relationship with OPN, including cathepsins B, L, S, and Z, GAL-3 (GAL-3 is normally a cytosolic protein recruited to damaged lysosomes and triggers selective autophagy [22]), Galectin-1, and CD44 (Figure 1A). Cathepsins B, L, S, and Z are a class of lysosomal cysteine proteases. Moreover, Figure 1B reveals that significantly enriched pathways in DEPs were identified using KEGG pathway analysis in the comparison between the HI and Sham groups, and KEGG pathway analysis suggested that the pathway activated by HI injury was associated with lysosomes. For this reason, we hypothesized that OPN might be connected to lysosomal function. First, we speculated whether OPN expression was upregulated by HI insult in neonatal mice and affected injury. Consistent with our previous finding [7], OPN expression was undetectable in the naïve neonatal brain and in the hemisphere contralateral to the lesion, but was gradually upregulated in the ipsilateral cortex at 3 days after HI (Figure 1C). Immunofluorescence staining showed that increased OPN expression was mainly found in the microglia/macrophages (intracellular OPN) of the ipsilateral cortex at 3 days following HI insult. A few granular OPNs (secreted OPN) were scattered among the amoeboid microglia/macrophages in the lesion core (Figure S1A). Moreover, OPN puncta in the microglia/macrophages were colocalized with the lysosomal marker LAMP1 and GAL-3 (Figure 1D,E).

To verify the findings in vivo, we also transfected HEK293T cells, which are easy to transfect and contain abundant lysosomes, with OPN-EGFP plasmid to overexpress OPN. The lysosome was stained with Lyso-Tracker Red and the mitochondria were stained with Mito-Tracker Red CMXRos. Similarly, the OPN-EGFP was colocalized with Lyso-Tracker Red (Figure 1F), not Mito-Tracker (Figure S1B). These data suggest that upregulated OPN may play an important role in lysosome function after HI insult.

### 3.2. HI Insult Led to Impairment of Lysosomal Function and Autophagic Flux

**Figure 2:**
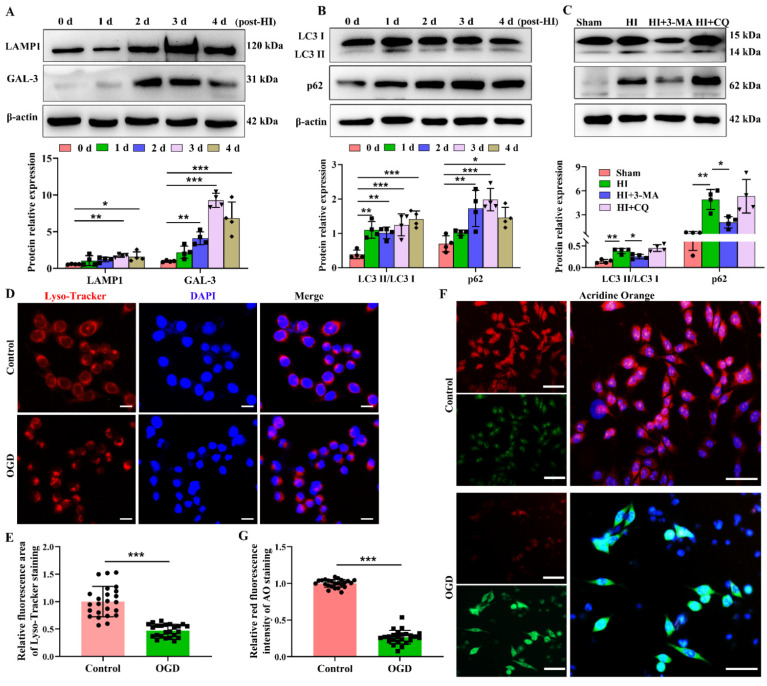
HI exposure led to lysosomal damage and disrupted autophagy flux. (**A**) Protein expression levels of LAMP1 and GAL-3 in the ipsilateral cortex were determined at 0 day (d), 1 d, 2 d, 3 d, and 4 d post-HI (*n* = 4). All experiments were performed three times. (**B**) The levels of LC3-II and p62 in the ipsilateral cortex were determined at 0 d, 1 d, 2 d, 3 d, and 4 d post-HI (*n* = 4). All experiments were performed three times. (**C**) The levels of LC3-II and p62 in the ipsilateral cortex with 3-MA or CQ treatment were determined at 3 d post-HI (*n* = 4). All experiments were performed three times. (**D**,**E**) BV-2 cells were incubated Lyso-Tracker Red to assess lysosomal stability following OGD (*n* = 6). (**F**,**G**) BV-2 cells were incubated with AO to evaluate lysosomal dysfunction following OGD (*n* = 6). Values represent the mean ± SD, * *p* < 0.05, ** *p* < 0.01, *** *p* < 0.001 according to one-way ANOVA with Dunnett corrections in (**A**,**B**); * *p* < 0.05, ** *p* < 0.01 according to one-way ANOVA with Bonferroni corrections in (**C**). *** *p* < 0.001 according to *t*-test in (**E**,**G**). 3-MA, 3-methyladenin; AO, acridine orange; CQ, chloroquine; DAPI, 4′,6′-diamidino-2-phenylindole dihydrochloride hydrate; GAL-3, galectin-3; HI, hypoxia–ischemia; LAMP1, lysosomal-associated membrane protein 1; LC3, microtubule-associated protein 1 light chain 3; OGD, oxygen–glucose deprivation.

Given that the lysosomal system plays an important role in homeostasis and neuronal integrity, we used different but complementary methods to evaluate lysosomal function. First, the effect of neonatal HI on the expression and distribution of some lysosomal proteins, such as LAMP1 (marker of lysosomes [23]) and GAL-3 was studied. The levels of LAMP1 mRNA and protein in the ipsilateral cortex were increased after HI exposure (Figure S2A and Figure 2A). The levels of GAL-3 mRNA and protein in the ipsilateral cortex were increased at 2 days and 3 days after HI exposure (Figure S2B and Figure 2A). Moreover, increased expression of GAL-3 was colocalized with LAMP1 (Figure S2C,D), similar to the previous report [24].

Lysosomes play an important role in autophagic degradation. Next, we examined the changes in the autophagic flux following HI insult. Consistent with our previous finding [25], the protein expression of microtubule-associated protein 1 light chain 3 (LC3) II in the ipsilateral cortex at 1 day, 2 days, 3 days, and 4 days post-HI increased significantly compared with the Sham group (Figure 2B). The protein expression of SQSTM1 (p62, a ubiquitin-binding protein delivered to lysosomes for degradation) gradually increased at 2 days, 3 days, and 4 days post-HI (Figure 2B), which suggested that the autophagic flux was significantly inhibited post-HI. We also found that 3-MA (inhibiting the formation of autophagosome precursors) effectively decreased the LC3-II and p62 levels at 3 days post-HI, indicating that the initiation of autophagy was affected by HI (Figure 2C). However, CQ (an inhibitor of autophagosome–lysosome fusion) could not elevate the LC3-II and p62 levels at 3 days post-HI (Figure 2C), indicating that its formation in the autolysosome was significantly blocked in HI brains and had remarkable ceiling effects.

Previous findings have shown that microglial autophagy plays an important pro-inflammatory role in cytokine production and the neuroinflammatory response after ischemic stroke. Hypoxia/ischemia could lead to the excessive activation of autophagy in microglia, which exacerbates neuroinflammatory damage [26]. Considering that OPN is mainly found in the microglia and is involved in inflammation [7], in this study, we focused on the role of genetic OPN in lysosomal damage and the autophagy of microglia/macrophages after neonatal stroke. Evidence of lysosomal damage was detected by using Lyso-Tracker Red and AO staining. In control BV-2 cells, lysosomes were uniformly distributed, while they accumulated in a perinuclear location in OGD-exposed BV-2 cells (Figure 2D,E). AO yielded red fluorescence when accumulated within the lysosome and green fluorescence when released from ruptured lysosomes and diffused into the cytosol and nuclei [10]. OGD exposure weakened the red fluorescence in BV-2 cells (Figure 2F,G). Consistent with the in vitro results, these data suggested that HI insult led to lysosomal dysfunction and a subsequent increase in autophagosome accumulation and autophagic degradation blockage.

### 3.3. OPN Deficiency Reduced HI-Induced Lysosomes Damage and Autophagosome Accumulation, Associating with Improving Behavior Deficit

**Figure 3:**
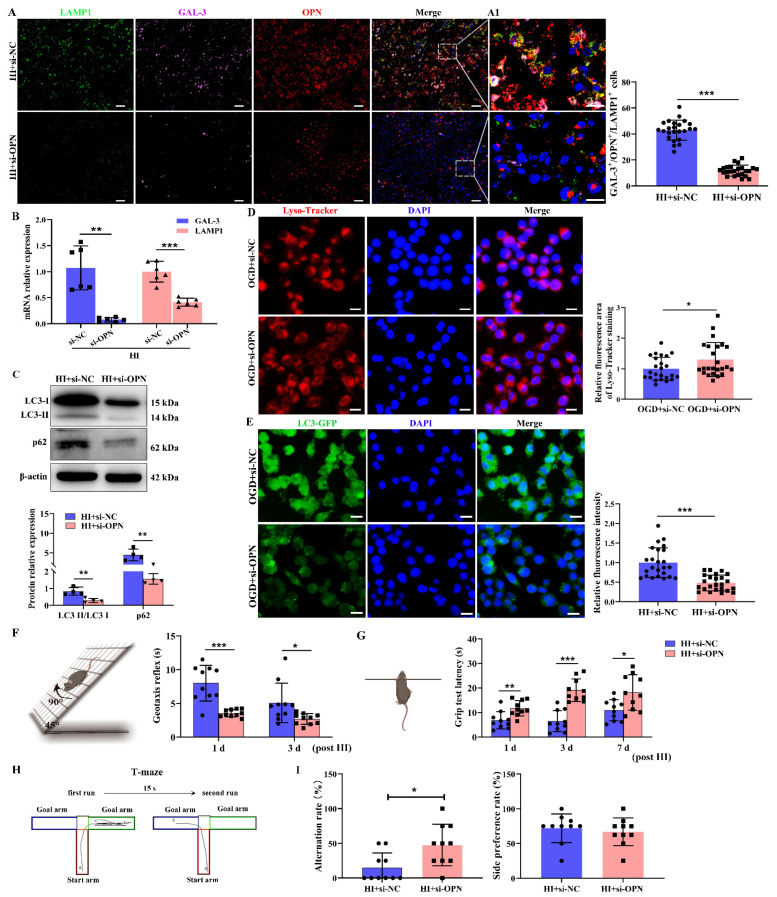
OPN deficiency attenuated lysosome damage and autophagosome accumulation after HI insult. (**A**) Immunofluorescence images of LAMP1, GAL-3, and OPN in the HI+si-OPN or HI+si-NC group (*n* = 4). Scale bar = 50 μm; A1: Magnified views of boxed regions in (**A**) showing the colocalization of LAMP1, GAL-3, and OPN. Scale bar = 20 μm. (**B**) GAL-3 and LAMP1 mRNAs in HI+si-NC or HI+si-OPN group were detected by qRT-PCR (*n* = 6). (**C**) The protein levels of LC3-II and p62 in the HI+si-NC or HI+si-OPN group were measured with Western blot (*n* = 4). All experiments were performed three times. (**D**) BV-2 cells were exposed to OGD after transfection with si-NC and si-OPN, then incubated with Lyso-Tracker Red (*n* = 6). Scale bar = 20 μm. (**E**) Immunofluorescence images of LC3-GFP after OGD in BV-2 cells transfected with si-NC and si-OPN (*n* = 4). Scale bar = 20 μm. (**F**) Geotaxis reflexes tests on 1 d and 3 d after HI exposure in the HI+si-NC and HI+si-OPN groups (*n* = 10). (**G**) Grip tests on 1 d, 3 d, and 7 d after HI exposure in the HI+si-NC and HI+si-OPN groups (*n* = 10). (**H**) Diagram of T-maze. (**I**) Alternation rate and side preference rate in HI+si-NC and HI+si-OPN groups (*n* = 10). Values represent the mean ± SD, * *p* < 0.05, ** *p* < 0.01, *** *p* < 0.001 according to the *t*-test. d, days; DAPI, 4′,6′-diamidino-2-phenylindole dihydrochloride hydrate; HI, hypoxia–ischemia; LAMP1, lysosomal-associated membrane protein 1; LC3, microtubule-associated protein 1 light chain 3; NC, negative control; OGD, oxygen–glucose deprivation; OPN, osteopontin.

To determine if OPN plays an important role in lysosome function, OPN was knocked down by lentiviral transfection in HI-injury mice (Figure S3A). Consistent with our previous finding [7], the genetic suppression of OPN significantly attenuated brain damage (Figure S3B–F). Upregulated LAMP1 and GAL-3 expressions in the ipsilateral cortex were reversed by the inhibition of OPN expression at 3 days following HI exposure (Figure 3A); meanwhile, the genetic suppression of OPN reduced HI-induced GAL-3 and LAMP1 mRNA expression (Figure 3B), demonstrating the attenuation in HI-induced damaged lysosome by OPN deficiency. In addition, blocking OPN expression decreased the LC3-II and p62 levels in the ipsilateral cortex at 3 days following HI exposure (Figure 3C). To assess whether OPN also follows a similar lysosomal function in vitro, the genetic suppression of OPN reversed OGD exposure-displaying a perinuclear location of Lyso-Tracker Red fluorescent signal (Figure 3D). The overexpression of OPN attenuated the red fluorescence of AO in BV-2 cells (Figure S3G). OGD exposure increased the LC3-GFP levels in the cytoplasm (Figure S3H,I), indicating that autophagosomes could not fully transform into autolysosomes, which was reversed by the knockdown of OPN with immunofluorescence staining (Figure 3E).

The potential effects of OPN silencing in neurological outcomes after HI insult were evaluated by geotaxis reflexes and grip test. Compared with HI+si-NC mice, the times for the geotaxis reflexes in the HI+si-OPN group were significantly shortened at 1 day and 3 days after HI (Figure 3F). In the assessment of the grip test, OPN deficiency significantly restored the grip strength at 1 day, 3 days, and 7 days post-injury (Figure 3G). These data suggested that OPN deficiency provided short-term protection against neonatal HI brain injury. We then sought to determine whether this beneficial effect was long-lasting by performing a T-maze test 28 days after HI injury. The results showed that OPN down-regulation increased the alternation rate compared with the HI+si-NC group (Figure 3H,I). However, the side preference rate in each group had no significant difference (Figure 3I).

### 3.4. OPN Deficiency Reduced CTSB Release into Cytoplasm and NLRP3 Inflammasome Activation after HI Insult

**Figure 4:**
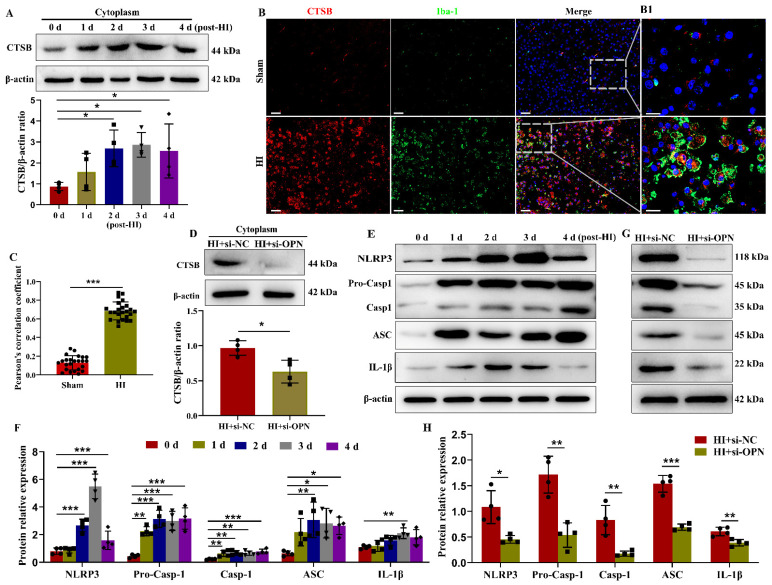
Silencing of OPN repressed HI-induced CTSB releasing and NLRP3 inflammasome activation. (**A**) The expression of CTSB protein in the cytoplasm of the ipsilateral cortex was measured by Western blot at 0 d, 1 d, 2 d, 3 d, and 4 d after HI (*n* = 4). All experiments were performed three times. (**B**,**C**) Immunofluorescence images and quantitative measurement revealed colocalization of CTSB and Iba-1 (*n* = 4). Scale bar = 50 μm; B1: Magnified views of boxed regions in (**B**) showing the colocalization of CTSB and Iba-1. Scale bar = 20 μm. (**D**) The protein expression levels of CTSB in the cytoplasm of the HI+si-NC or HI+si-OPN group were determined by Western blott (*n* = 4). All experiments were performed three times. (**E**,**F**) NLRP3, Pro-Caspase-1 (Pro-Casp-1), Caspase-1 (Casp-1), ASC, and IL-1β proteins in the ipsilateral cortex were assessed at 0 d, 1 d, 2 d, 3 d, and 4 d post-HI by Western blot (*n* = 4). All experiments were performed three times. (**G**,**H**) NLRP3, Pro-Casp-1, Casp-1, ASC, and IL-1β proteins in the HI+NC or HI+si-OPN group were examined by Western blot (*n* = 4). All experiments were performed three times. Values represent the mean ± SD, * *p* < 0.05, ** *p* < 0.01, *** *p* < 0.001 according to the *t*-test in (**C**,**D**), and (**H**); * *p* < 0.05, ** *p* < 0.01, *** *p* < 0.001 according to one-way ANOVA with Dunnett corrections in (**A**,**F**). ASC, apoptosis-associated speck-like protein containing a CARD; CTSB, cathepsin B; HI, hypoxia–ischemia; Iba-1, ionized calcium-binding adapter molecule 1; IL-1β, interleukin-1β; LAMP1, lysosomal-associated membrane protein 1; NC, negative control; NLRP3, NOD-like receptor thermal protein domain-associated protein 3; OPN, osteopontin.

We next investigated whether the lysosome dysfunction induced by HI resulted in the release of CTSB into the cytosol. The expressions of CTSB mRNA and protein in the cytosol were increased following HI insult (Figure S4A and Figure 4A). The increased expression of CTSB was primarily in the Iba1^+^ microglia/macrophages, NeuN^+^ neurons, and GFAP^+^ astrocytes in the ipsilateral cortex at 3 days following HI (Figure 4B,C and Figure S4B–D). Moreover, the genetic suppression of OPN attenuated the HI-upregulated CTSB protein in the cytoplasm and mRNA of the ipsilateral cortex at 3 days following HI exposure (Figure 4D and Figure S4E), indicating that HI-induced damaged lysosomes and release of CTSB were reduced after OPN deficiency.

The leakage of CTSB from the lysosomes to the cytoplasm can trigger the activation of the NLRP3 inflammasome in microglia/macrophages [12]. Next, we examined whether the up-regulation of OPN in microglia/macrophages might trigger the activation of the NLRP3 inflammasome and promote the production of IL-1β. The levels of NLRP3, Caspase-1, apoptosis-associated speck-like protein containing a CARD (ASC), and IL-1β mRNAs and proteins were up-regulated in the ipsilateral cortex after HI exposure (Figure S5A and Figure 4E,F). Consistent with the importance of OPN in the regulation of CTSB expression, the genetic suppression of OPN concurrently reduced the levels of NLRP3, Caspase-1, ASC, and IL-1β mRNA and protein in the ipsilateral cortex following HI exposure (Figure S5B and Figure 4G,H), demonstrating that OPN plays a critical role in NLRP3 inflammasome activation in microglia/macrophages.

### 3.5. OPN Interacts with GAL-3 to Induced Lysosomal Damage following HI Exposure

**Figure 5:**
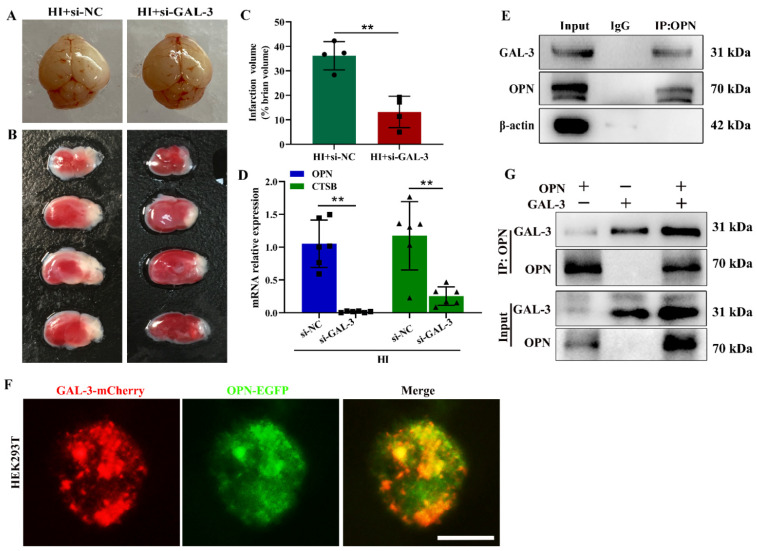
OPN interacted with GAL-3 in microglia/macrophages following HI exposure. (**A**) Representative images of brains at 3 d following HI treated with si-GAL-3 or si-NC (*n* = 4). (**B**,**C**) Representative TTC staining and quantification of brains infarct volumes at 3 d following HI treated with si-GAL-3 or si-NC (*n* = 4). (**D**) OPN and CTSB mRNAs in the HI+si-NC or HI+si-GAL-3 group were detected by qRT-PCR (*n* = 6). (**E**) Western blot showing co-IP of OPN with GAL-3 in BV-2 cells treated with OGD for 3 h. All experiments were performed three times. (**F**) Fluorescence analysis showed that OPN was colocalized with GAL-3 in HEK293T cells transfected with plasmids encoding OPN-EGFP together with GAL-3-mCherry (*n* = 6). (**G**) Co-IP assays of OPN interacting with GAL-3 in vitro. All experiments were performed three times. Scale bar = 20 μm. Values represent the mean ± SD, ** *p* < 0.01 according to the *t*-test in (**C**,**D**). co-IP, co-immunoprecipitation CTSB, cathepsin B; GAL-3, galectin-3; HI, hypoxia–ischemia; NC, negative control; OPN, osteopontin.

As mentioned above, GAL-3, β-galactoside binding cytosolic lectin, has been shown to play a critical role in autophagy responses to lysosomal damage [27], and the genetic suppression of OPN reduced HI-induced GAL-3 expression in the ipsilateral cortex (Figure 3B). Next, we examined whether the up-regulation of OPN in the microglia/macrophages might interact with GAL-3 to induce lysosomal damage following HI insult. Blocking GAL-3 expression could alleviate HI-induced brain injury (Figure 5A–C) and HI-induced OPN and CTSB mRNA expression in the ipsilateral cortex (Figure 5D).

The co-IP experiments were performed to determine the interaction between OPN and GAL-3. IP with OPN antibody using cell lysates from BV-2 cells treated with OGD found that endogenous OPN and GAL-3 formed a complex, indicating that there was a direct or indirect interaction between OPN and GAL-3 (Figure 5E). In order to observe this binding effect more intuitively, we co-transfected GAL-3-mcherry and OPN-EGFP plasmids in HEK293T cells, and the fluorescence results showed that GAL-3 was colocalized with OPN (Figure 5F). Then, in order to further explore whether GAL-3 and OPN were directly bound, we obtained GAL-3 and OPN protein translation using TnT^®^ T7 Coupled Reticulocyte Lysate Systems in vitro and conducted co-IP experiments. The results found that there was a direct interaction between GAL-3 and OPN in vitro (Figure 5G). The above results indicated that OPN and GAL-3 directly interacted to induce lysosomal damage in microglia/macrophages.

### 3.6. Cleaved OPN Promoted Lysosomal Damage by Interacting with GAL-3

**Figure 6:**
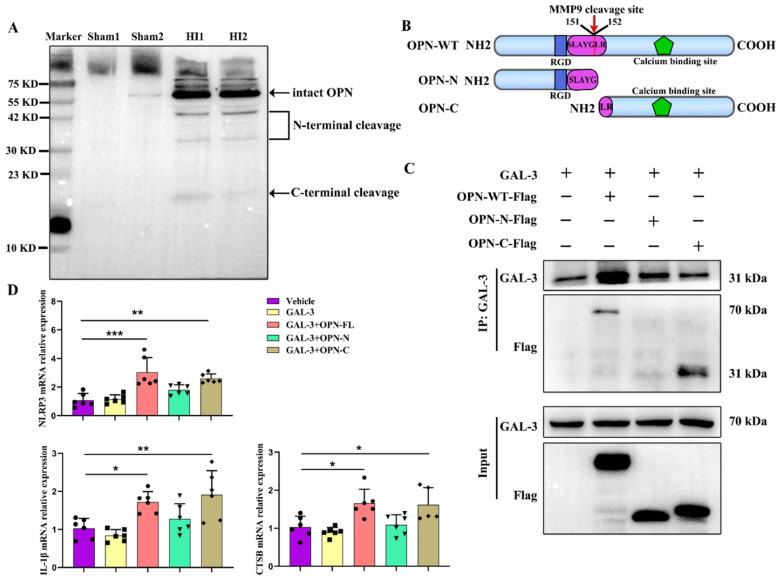
Cleaved OPN promotes lysosomal damage by interacting with GAL-3. (**A**) Western blot analysis of OPN in the ipsilateral cortex at 3 d using anti-OPN antibody. All experiments were performed three times (*n* = 4). (**B**) Truncated versions of OPN shown schematically, including N-terminal fragment and C-terminal fragment. (**C**) Co-IP assays detecedthe interaction of GAL-3 with OPN-WT-Flag, OPN-N-Flag, and OPN-C-Flag. All experiments were performed three times. (**D**) NLRP3, IL-1β, and CTSB mRNAs were assessed by qRT-PCR (*n* = 6). Values represent the mean ± SD, * *p* < 0.05, ** *p* < 0.01, *** *p* < 0.001 according to one-way ANOVA with Bonferroni corrections in (**D**). OPN-C, OPN-C-terminal fragment; co-IP, co-immunoprecipitation; CTSB, cathepsin B; GAL-3, galectin-3; HI, hypoxia–ischemia; IL-1β, interleukin-1β; LAMP1, lysosomal-associated membrane protein 1; NLRP3, NOD-like receptor thermal protein domain-associated protein 3; OPN-N, OPN-N-terminal fragment; OPN, osteopontin; WT, wild-type.

The biological activity of OPN can be modulated by proteolytic cleavage in the microenvironment and has been shown to be a substrate for thrombin and matrix metalloproteinases (MMP) [28,29]. First, we found that the levels of MMP-9 mRNA were greatly elevated at 3 days post-HI (Figure S6), confirming earlier results [30]. We found a significantly increased intensity of the full-length OPN band at ∼70 kDa, as well as three significantly increased fragments migrating at ∼20 kDa, ∼30 kDa, and ∼42 kDa detected by murine OPN antibody (Figure 6A).

Next, we investigated whether cleaved OPN and intact OPN could bind GAL-3 and promote lysosome damage. Two OPN truncations, including the N-terminal fragment (Met1-Gly151) and the C-terminal (Leu152-Asn294) fragment, were constructed and expressed in vitro (Figure 6B). Plasmids of GAL-3, OPN-WT-Flag, OPN-N-Flag, and OPN-C-Flag were constructed and used to infect HEK293T cells. As shown in Figure 6C, OPN-WT and OPN-C, but not OPN-N, were bound to GAL-3. Further, we investigated the roles of OPN cleavage products in neuroinflammation by using OPN-N and OPN-C. The results showed that OPN-WT or OPN-C with GAL-3 overexpression up-regulated NLRP3, IL-1β, and CTSB mRNA expression in BV-2 cells. However, OPN-N had no effect (Figure 6D). These data indicated that WT-OPN and cleaved OPN-C bound to GAL-3, leading to lysosomal damage.

### 3.7. Role of CD44 in OPN-Induced Lysosomal Damage following HI Insult

**Figure 7:**
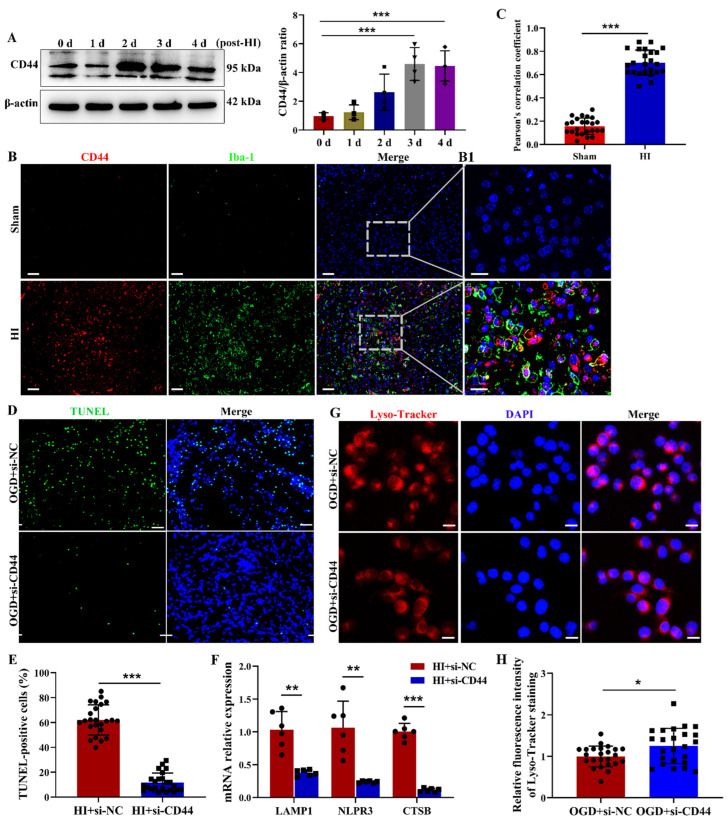
Role of CD44 in HI-induced lysosome leakage and CTSB release. (**A**) CD44 protein in the ipsilateral cortex was assessed at 0 d, 1 d, 2 d, 3 d, and 4 d post-HI by Western blot (*n* = 4). All experiments were performed three times. (**B**,**C**) Immunofluorescence images and quantitative measurements revealed the colocalization of CD44 and Iba-1 (*n* = 4). Scale bar = 50 μm. B1: Magnified views of the boxed regions in (**B**) showing the colocalization of CD44 and Iba-1. Scale bar = 20 μm. (**D**,**E**) TUNEL staining at 3 d following HI-injured mice pre-treated with si-CD44 or si-NC (*n* = 4). Scale bar = 50 μm. (**F**) LAMP1, NLRP3, and CTSB mRNAs in HI+si-NC or HI+si-OPN group were assessed by qRT-PCR (*n* = 6). (**G**,**H**) BV-2 cells were exposed to OGD after transfection with si-NC and si-OPN, followed by incubation with Lyso-Tracker Red (*n* = 6). Scale bar = 20 μm. Values represent the mean ± SD, *** *p* < 0.001 according to one-way ANOVA with Dunnett corrections in (**A**). * *p* < 0.05, ** *p* < 0.01, *** *p* < 0.001 according to the *t*-test in (**C**,**E**,**F**,**H**). CTSB, cathepsin B; DAPI, 4′,6′-diamidino-2-phenylindole dihydrochloride hydrate; HI, hypoxia–ischemia; Iba-1, ionized calcium-binding adapter molecule 1; LAMP1, lysosomal-associated membrane protein 1; NC: negative control; NLRP3, NOD-like receptor thermal protein domain associated protein 3; TUNEL, terminal deoxynucleotidyltransferase-mediated dUTP-biotin nick end labeling; OGD, oxygen–glucose deprivation.

CD44, αv, and β_3_ are the most well-characterized integrin receptors for OPN. The induction of CD44 was restricted to activated microglia/macrophages within sites of intense neural damage in the ischemic brain [31]. In addition, αv and β_3_ integrin subunits were strongly induced in reactive astrocytes [32]. We found that the levels of CD44 mRNA and protein in the ipsilateral cortex were increased after HI exposure (Figure S7A and Figure 7A). Double labeling analysis indicated that CD44 immunoreactivity was mainly localized in large, round amoeboid-like brain microglia/macrophages (Figure 7B,C). In the ipsilateral cortex, a few NeuN^+^ neuron cells and GFAP^+^ astrocytes expressed CD44 (Figure S8). In addition, the mRNA level of αv integrin subunits in the ipsilateral cortex increased at 2 days post-HI, while the mRNA level of β_3_ integrin subunits in the ipsilateral cortex decreased at 3 days after HI (Figure S7B). As αv and β_3_ integrin subunits were strongly induced in reactive astrocytes after ischemia, CD44 was supposed to be the main OPN receptor in microglia/macrophages following HI in neonatal mice.

To determine whether CD44 mediated OPN-induced lysosomal dysfunction following HI exposure, mice were pre-treated with si-CD44 to suppress CD44 expression. The CD44 deficiency inhibited HI-induced brain damage (Figure 7D,E and Figure S9) and the mRNA levels of LAMP1, NLRP3, and CTSB (Figure 7F). The genetic suppression of CD44 also reversed OGD exposure-displaying a perinuclear location of Lyso-Tracker Red fluorescent signal in BV-2 cells (Figure 7G,H). Moreover, blocking CD44 with antibody also attenuated HI-induced edema and infarct volume (Figure S10A–C). The inhibition of CD44 expression reduced CTSB and NLRP3 mRNA levels (Figure S10D,E). These data demonstrated that CD44 played a critical role in lysosomal dysfunction and CTSB release in the microglia.

### 3.8. Secreted OPN Stimulated Pro-Inflammatory Cytokines and CTSB Release through Binding with Cell Surface Receptors CD44

**Figure 8:**
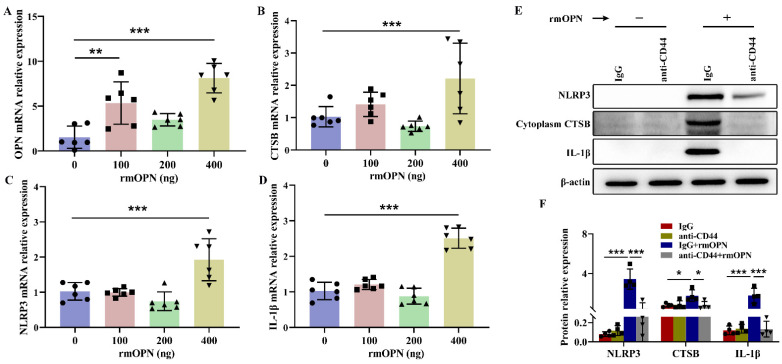
Secreted OPN regulated the inflammatory response by interaction with CD44 in vitro. (**A**–**D**) qRT-PCR was used to determine the expressions of OPN, CTSB, NLRP3, and IL-1β in BV-2 cells incubated with rmOPN (100, 200, and 400 ng/mL) (*n* = 6). (**E**,**F**) Western blot analysis of NLRP3, cytoplasmic CTSB, and IL-1β to 24 h of IgG or anti-CD44 treatment with/without rmOPN (400 ng/mL) treatment in BV-2 cells (*n* = 4). All experiments were performed three times. Values represent the mean ± SD, ** *p* < 0.01, *** *p* < 0.001 according to one-way ANOVA with Dunnett correction in (**A**–**D**). * *p* < 0.05, *** *p* < 0.001 according to one-way ANOVA with Bonferroni corrections in (**F**). CTSB, cathepsin B; IL-1β, interleukin-1β; NLRP3, NOD-like receptor thermal protein domain-associated protein 3; OPN, osteopontin; rmOPN, recombinant mouse OPN.

Next, we determined whether secreted OPN played a role in lysosomal dysfunction through binding with CD44. BV-2 cells were co-incubated with recombinant mouse OPN (rmOPN), and the qRT-PCR results showed that the expression of OPN mRNA was significantly up-regulated at the concentrations of 100 and 400 ng/mL rmOPN (Figure 8A). At the same time, the levels of CTSB, NLRP3, and IL-1β mRNA were up-regulated at the concentration of 400 ng/mL rmOPN in BV-2 cells (Figure 8B–D).

The total cellular RNA was extracted from BV-2 cells with antibodies against CD44 or IgG and treated with/without rmOPN (No decrease in CD44 mRNA levels was observed following transfection with si-CD44 in BV-2 cells in the preliminary experiment, probably because of the low levels of CD44 in resting microglia. Herein we used CD44 antibody to block CD44 function). The Western blot results showed that anti-CD44 reversed the significant rmOPN-induced increases in the NLRP3, CTSB, and IL-1β levels in BV-2 cells (Figure 8E,F). These results suggested that secreted OPN bound to cell surface receptors CD44 and stimulated CTSB release and inflammation in BV-2 cells.

## 4. Discussion

The present study demonstrated the vital role of OPN in HI-mediated lysosomal damage and autophagosome accumulation. We found that, at the early stage of ischemia, OPN expression was enhanced, especially in microglia, and colocalized with LAMP1 and GAL-3, which was accompanied by lysosomal damage, CTSB release, NLRP3 inflammasome activation, and autophagosomes accumulation after HI insult. Importantly, the knockdown of OPN expression significantly rescued the lysosomal damage with significant improvements in the autophagic flux following HI insult in neonatal mice, thus alleviating the HI brain damage. We identified, for the first time, the presence of OPN cleavage activity in the brain following HI insult. OPN and its cleavage product interacted with GAL-3, and secreted OPN combined with CD44 led to lysosomal damage and exacerbated autophagosome accumulation after HI exposure.

Lysosomal dysfunction, as reflected by cytosolic acidification and rupture/permeabilization, was detected after ischemic insult [33]. Cathepsins play an important role in a range of cellular activities, such as protein degradation, antigen presentation, and cell death [34]. Several lines of evidence suggest that the leakage of CTSB from the lysosomes into the cytoplasm could activate the NLRP3 inflammasome and affect the processing and secretion of pro-inflammatory cytokines [35,36,37]. In the present study, we found that acidic compartments accumulated and the expression of lysosomal markers (LAMP1, GAL-3, and CTSB) was higher in the ipsilateral cortex of HI mice, suggesting the existence of a disruption of the lysosomal compartment in HI animals.

Autophagy terminates with the degradation of the autophagosome contents in the lysosomes [38,39]. Our results demonstrated that HI insult led to a significant increase in the ratio of LC3-II to LC3-I over time. Simultaneously, the up-regulation of p62 was accompanied by an increase in autophagosomes, indicating that autophagic flux was inhibited post-HI. The increase in LC3-II could have been due to either the excessive initiation of autophagy or poor lysosomal clearance. We observed defects in lysosomal function after HI injury; thus, it is likely that the accumulation of LC3II in our study was due to lysosomal dysfunction. Therefore, the improvement of lysosomal dysfunction and autophagic flux are the current targets for the development of HI brain-damage drugs. Here, the PPI network showed that lysosomal cysteine proteases (cathepsins B, L, S, and Z, galectin-1, and GAL-3) exhibited potential relationships with OPN. Moreover, LAMP1, CTSB, GAL-3 proteins, and respective mRNAs were significantly increased and expressed in the lysosomes of microglia/macrophages in the brain after HI. OPN deficiency abrogated the processing and release of CTSB and NLRP3 inflammasome activation in HI-exposed microglia, as well as recovered impaired autophagic flux, suggesting that an increase in OPN in the microglia could cause defects in lysosomal function and abnormal autophagosome accumulation.

Within the brain, GAL-3 is expressed by microglia and some astrocytes, and weakly in some cortical neurons [40]. The extracellular or intracellular GAL-3 levels are elevated in a variety of pathologies, potentially due to neuroinflammation [41,42]. GAL-3 modulates the inflammatory response of the nervous system and has been implicated in the pathogenesis of diverse neurological diseases, such as models of stroke [43,44] and neonatal HI brain injury [45,46]. Extracellular GAL-3 could activate microglia by directly activating TLR4 [47]. The genetic deletion of GAL-3 protected against HI injury, particularly in the hippocampus and striatum in neonatal mice [48]. In adult Huntington’s disease mice, the up-regulation of GAL-3 formed puncta in damaged lysosomes in primary microglia and contributed to inflammation through NF-κB and NLRP3 inflammasome-dependent pathways [24]. Consistent with these previous findings, we found, in the present study, that the down-regulation of GAL-3 in the HI-injured mice not only attenuated brain damage, but also reduced HI-induced OPN and CTSB expression. Moreover, the co-IP result demonstrated a direct protein–protein interaction between OPN and GAL-3. The suppression of OPN was able to reduce HI-induced GAL-3 expression. Collectively, these observations suggest that intracellular OPN played a role in lysosomal damage through the association of OPN with GAL-3 in microglia.

It is noteworthy that OPN is not a sole cytokine, but rather a compound arrangement of multiple peptides that includes splice variants and several active proteolytic cleavage products. For example, OPN cleaved by MMP forms the OPN-N-terminal fragment and the OPN-C terminal fragment, the molecular weights of which are approximately ~40 kDa, ~32 kDa, and ~25 kDa, respectively [49]. Recent studies have found that the MMP-3 or 7 cleavage of OPN-C fragments results has aα9β1 binding site, which facilitates its role in the development of inflammatory arthritis [50]. Another study has shown that OPN-C promotes inflammation by the activation of the NF-κB pathway [51]. We found that OPN-WT and cleaved forms of OPN coexisted in the ipsilateral cortex following HI injury in neonatal mice, and the C-terminal domain of OPN was the molecular basis for the direct binding between OPN and GAL-3. WT-OPN and the cleavage of OPN increased pro-inflammatory cytokines and CTSB release. Taken together, our data indicate an amplifying loop of OPN and GAL-3 in HI-associated lysosomal damage that drives autophagosome accumulation and brain injury.

OPN is known to be involved in various pathophysiological events and has been studied as a secreted protein (secreted OPN). Secreted OPN can bind to multiple integrins, such as αvβ3, αvβ5, αvβ1, and α5β1, and to certain variant forms of CD44 [52]. OPN has multiple effects in different cell types, with distinct outcomes for disease phenotypes because of these varied receptors. For instance, OPN regulates hepatitis C virus replication and assembly by binding to the receptors αVβ3 and CD44 [53]. OPN-upregulated cyclooxygenase-2 expression in tumor-associated macrophages leads to enhanced angiogenesis and tumor growth via α9β1 integrin [54]. Macrophage-secreted OPN binds to CD44 on the tumor cells and promotes tumor invasion and clonal growth [55]. In the current study, we observed that secreted OPN dramatically up-regulated NLRP3 IL-1β and CTSB expression. Small interfering-mediated knockdown and antibody neutralization experiments identified CD44 as the OPN receptor that stimulated CTSB expression and NLRP3 inflammasome activation in microglia. Collectively, our observations indicated a critical role for secreted OPN in microglia-mediated neuroinflammation through binding to CD44.

## 5. Conclusions

There were also some limitations in our study. Firstly, lysosomal membrane destabilization may lead to the release of various lysosomal cysteine proteases into the cytosol. There might be other signaling factors that are affected by OPN. Second, several studies, including our previous study, showed that activated microglia/macrophages are the main cellular sources of OPN [7]; therefore, the effects of OPN deficiency on neuroinflammation in this study were examined by shRNA. In the future, the use of microglia-specific depletion of OPN mice might be considered to accurately measure the effects of OPN deficiency on neuroinflammation. Thirdly, in the current study, we were able to detect the band corresponding to the cleaved form without the need for any artificial digestion. The commercial murine antibodies directed against the OPN-N-terminal fragment or OPN-C-terminal fragment are not available, and we did not specifically detect OPN cleavage. Finally, the role of OPN cleavage in HI injury has not been investigated yet. Furthermore, the underlying mechanisms and modulating signals are poorly understood.

A striking observation of our studies is the regulation of lysosomal function by endogenous OPN, as well as secreted forms of OPN through CD44-mediated signaling pathways. We demonstrated the potential involvement of intracellular OPN in lysosomal damage and autophagic flux through the interaction of OPN and/or OPN cleavage with GAL-3 in the microglia. Secreted OPN was able to bind to microglial CD44 in an autocrine/paracrine manner and induce lysosomal damage. The knockdown of OPN expression could restore lysosomal damage with significant improvement in the autophagic flux and protect the brain against HI insult.

## Figures and Tables

**Table 1:** Antibody information.

Description	Catalog	Company	Dilution	
	Number		IF	WB
Mouse β-actin antibody	TA-09	Zhongshan Golden Bridge Biotechnology		1:1000
Rabbit OPN antibody	22952-1-AP	Proteintech	1:200	1:1000
Mouse GAL-3 antibody	sc-32790	Santa Cruz	1:200	1:1000
Rabbit NLPR3 antibody	ab263899	Abcam		1:1000
Rabbit CTSB antibody	12216-1-AP	Proteintech	1:200	1:1000
Rabbit LAMP1 antibody	ab24170	Abcam		1:1000
Rabbit IL-1β antibody	sc-7884	Santa Cruz		1:1000
Mouse NeuN antibody	ab104224	Abcam	1:200	
Mouse Iba-1 antibody	66827-1-Ig	Proteintech	1:200	
Mouse GFAP antibody	60190–1-Ig	Proteintech	1:200	
Rabbit LC3B antibody	#2775	Cell Signaling Technology		1:1000
Rabbit p62 antibody	18420-1-AP	Proteintech		1:1000
Rabbit CD44 antibody	15675-1-AP	Proteintech	1:200	1:1000
Mouse Flag M2 antibody	F1802	Sigma-Aldrich		1:1000
Rabbit Caspase-1 antibody	GB11383	Servicebio		1:1000
Rabbit ASC antibody	GB113966	Servicebio		1:1000
Goat OPN antibody	ab11503	Abcam	1:200	

**Table 2:** Primer information.

Accession Number	Genes	Primer Sequence (5′–3′)
NM_00141184 3.1	GAPDH (112 bp)	F primer: ATACGGCTACAGCAACAGGG R primer: GCCTCTCTTGCTCAGTGTCC
NM_023258.4	ASC (106 bp)	F primer: CTAGTTTGCTGGGGAAAGAAC R primer: CTAAGCACAGTCATTGTGAGCTC
NM_009807.2	Caspase-1 (72 bp)	F primer: CGTACACGTCTTGCCCTCAT R primer: AACTTGAGCTCCAACCCTCG
XM_006498- 649.2	CD44 (191 bp)	F primer: ATGAAGTTGGCCCTGAGCAA R primer: TCTTCTTCAGGAGGGGCTGA
NM_007798.3	CTSB (103 bp)	F primer: GCAGCCAACTCTTGGAACCTT R primer: GGATTCCAGCCACAATTTCTG
NM_010705.3	Galectin-3 (73 bp)	F primer: TTGAAGCTGACCACTTCAAGGTT R primer: AGGTTCTTCATCCGATGGTTGT
NM_008361.4	IL-1β (165 bp)	F primer: CTCACAAGCAGAGCACAAGC R primer: AGCTGTCTGCTCATTCACGA
NM_001317- 353.1	LAMP1 (88 bp)	F primer: TCGTGAACATTTCCCTGCCA R primer: GTGAGGCTGGGGTCAGAAAC
XM_00653075 1.4	MMP-2 (96 bp)	F primer: CCTGGACCCTGAAACCGTG R primer: TCCCCATCATGGATTCGAGAA
NM_0013199- 86.1	MMP-7 (93 bp)	F primer: CTTACCTCGGATCGTAGTGGA R primer: CCCCAACTAACCCTCTTGAAGT
NM_004994.3	MMP-9 (149 bp)	F primer: AAGGGTACAGCCTGTTCCTGGT R primer: CAGGATGCCGTCTATGTCGTC
NM_145827.4	NLRP3 (83 bp)	F primer: ACGAGTCCTGGTGACTTTGTAT R primer: TAGGTCCACACAGAAAGTTCTCTTA
NM_001204- 203.1	OPN (221 bp)	F primer: AGCCACAAGTTTCACAGCCACAAG R primer: CTGAGAAATGAGCAGTTAGTATTC

## Data Availability

The datasets generated during the current study are available from the corresponding author upon reasonable request.

## Supplementary Materials

The following supporting information can be downloaded at: https://www.mdpi.com/article/10.3390/cells12060854/s1, Figure S1: Immunofluorescence staining to analyzed the cellular distribution of OPN; Figure S2: The mRNA levels of LAPM1 and CTSB were up-regulated after HI insult; Figure S3: Silence of OPN attenuated brain damage after HI insult; Figure S4: CTSB was localized in NeuN+ and GFAP+ cells; Figure S5: Change of mRNA levels of NLRP3 inflammasome; Figure S6: The MMP9 expression was observed post-HI; Figure S7: The CD44, αv and β3 mRNA expression was observed post-HI; Figure S8: CD44 was colocalized in NeuN+ and GFAP+ cells; Figure S9: Silence of CD44 attenuated brain damage after HI insult; Figure S10: Anti-CD44 blocked HI induce brain damage and lysosomal.

## Abbreviations

3-MA	3-Methyladenin
AO	Acridine orange
ASC	Apoptosis-associated speck-like protein containing a CARD
co-IP	Co-immunoprecipitation
CQ	Chloroquine
CTSB	Cathepsin B
DAPI	4′,6′-Diamidino-2-phenylindole dihydrochloride hydrate
DEPs	Differentially expressed proteins
GAL-3	Galectin-3
GFAP	Glial fibrillary acidic protein
HI	Hypoxia–ischemia
Iba-1	Ionized calcium-binding adapter molecule 1
IL-1β	Interleukin-1β
LAMP1	Lysosomal-associated membrane protein 1
LC3	Microtubule-associated protein 1 light chain 3
MMP	Matrix metalloproteinases
NC	Negative control
NLRP3	NOD-like receptor thermal protein domain associated protein 3
NeuN	Neuronal specific nuclear protein
OGD	Oxygen–glucose deprivation
OPN	Osteopontin
qRT-PCR	Quantitative real-time polymerase chain reaction
rmOPN	Recombinant mouse OPN
TUNEL	Terminal deoxynucleotidyltransferase-mediated dUTP-biotin nick end labeling
TTC	2,3,5-Triphenyltetrazolium chloride monohydrate
