# Supplementary material for: Mechanistic Insights into the Role of OPN in Mediating Brain Damage via Triggering Lysosomal Damage in Microglia/Macrophage
Source: Cells. 2023 Mar 9;12(6):854. doi: 10.3390/cells12060854 (PMC10046941; doi:10.3390/cells12060854)
Supplement: Supplementary file 1 [file cells-12-00854-s001.zip › cells-2160755-supplementary.pdf]

Figure supplement data

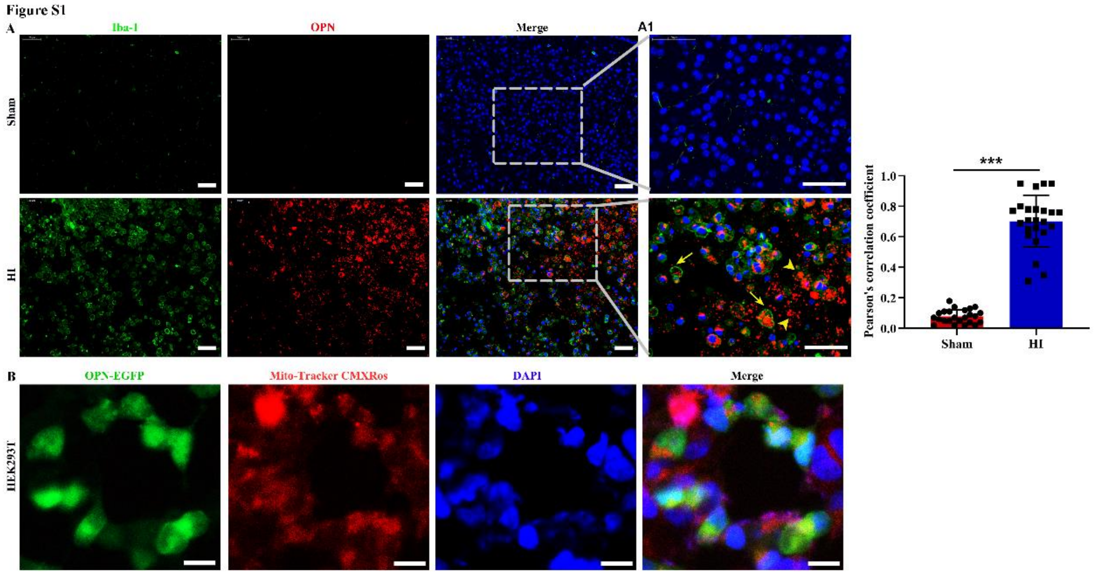

Figure S1. Immunofluorescence staining to analyzed the cellular distribution of OPN. (**A**) OPN was upregulated and localized to microglia/macrophages. Scale bar = 50 μm; A1: Magnified views of boxed regions in A showing colocalization of OPN and Iba-1 (n=4). Scale bar = 20 μm. (**B**) HEK293T cells were stably transfected with OPN-mcherry, and labeled with Mito-Tracker (n=6). Scale bar = 20 μm. Values represent the mean ± SD, ***$p < 0.001$ according to t-test in (**A**). DAPI, 4′,6′-diamidino-2-phenylindole dihydrochloride hydrate; HI, hypoxia-ischemia; Iba-1, ionized calcium binding adapter molecule 1; OPN, osteopontin.

Figure S2

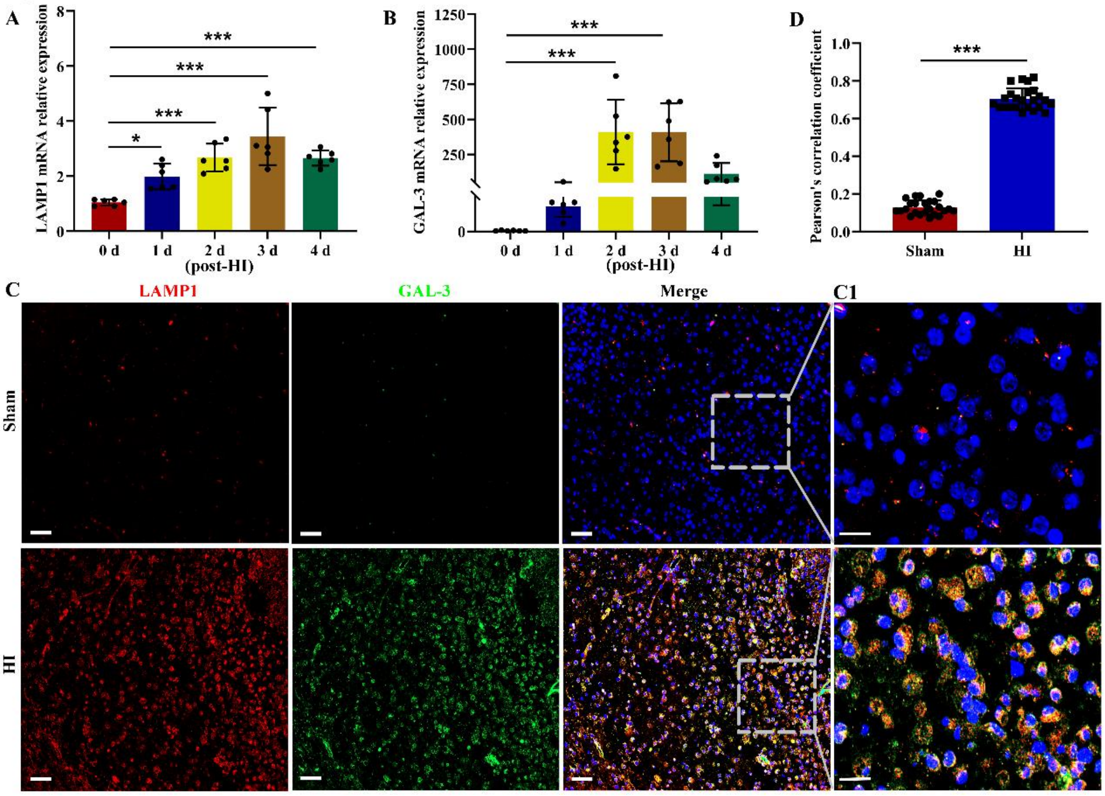

Figure S2. The mRNA levels of LAPM1 and CTSB were up-regulated after HI insult. (**A**)

Expression of LAMP1 mRNA in ipsilateral cortex was detected at 0 d, 1 d, 2 d, 3 d and 4 d post-HI (n=6). (**B**) Expression of CTSB mRNA in ipsilateral cortex was detected at 0 d, 1 d, 2 d, 3 d and 4 d post-HI (n=6). (**C,D**) GAL-3 was localized in LAMP1 in brain. Scale bar = 50 μm; C1: Magnified views of boxed regions in C showing colocalization of GAL-3 and LAMP1 (n=4). Scale bar = 20 μm. Values represent the mean ± SD, *$p < 0.05$, ***$p < 0.001$ according to one-way ANOVA with Dunnett corrections in (**A**) and (**B**); Values represent the mean ± SD, ***$p < 0.001$ according to *t*-test in D. d, days; GAL-3, galectin-3; HI, hypoxia-ischemia; LAMP1, lysosomal-associated membrane protein 1.

Figure S3

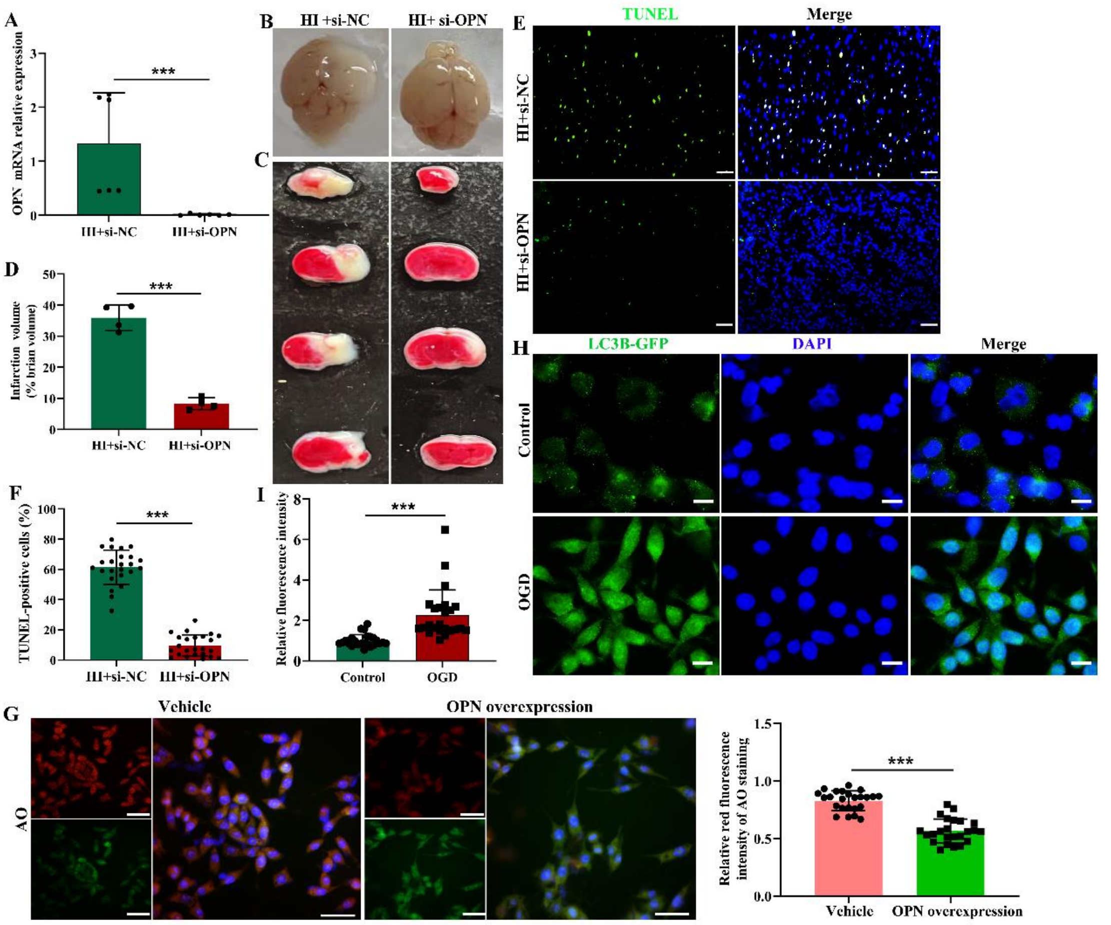

Figure S3. Silence of OPN attenuated brain damage after HI insult. (**A**) The OPN mRNA level was detected after transfection with si-NC, si-OPN in HI-injured mice (n=6). (**B**) Representative brain pictures at 3 d following HI treated with si-OPN or si-NC (n=4). (**C**) Example of images of TTC staining at 3 d following HI treated with si-OPN or si-NC (n=4). (**D**) Quantification of brain infarct volume at 3 d following HI treated with si-NC or si-OPN (n=4). (**E,F**) TUNEL staining at 3 d following HI treated with si-NC or si-OPN (n=4). Scale bar = 50 μm. (**G**) BV-2 cells were incubated with AO to evaluated lysosomal dysfunction after overexpression of OPN (n=6). (**H,I**) Immunofluorescence images of LC3-GFP in BV2 cells with or without OGD (n=6). Scale bar = 20 μm. Values represent the mean ± SD, ***$p < 0.001$ according to *t*-test in (**A,D,F,G**) and (**I**). d, days; HI, hypoxia-ischemia; NC, negative control; OGD, Oxygen–glucose deprivation; OPN, osteopontin; qRT-PCR, quantitative real time polymerase chain reaction; TUNEL, terminal deoxynucleotidyltransferase-mediated

dUTP-biotin nick end labeling.

Figure S4

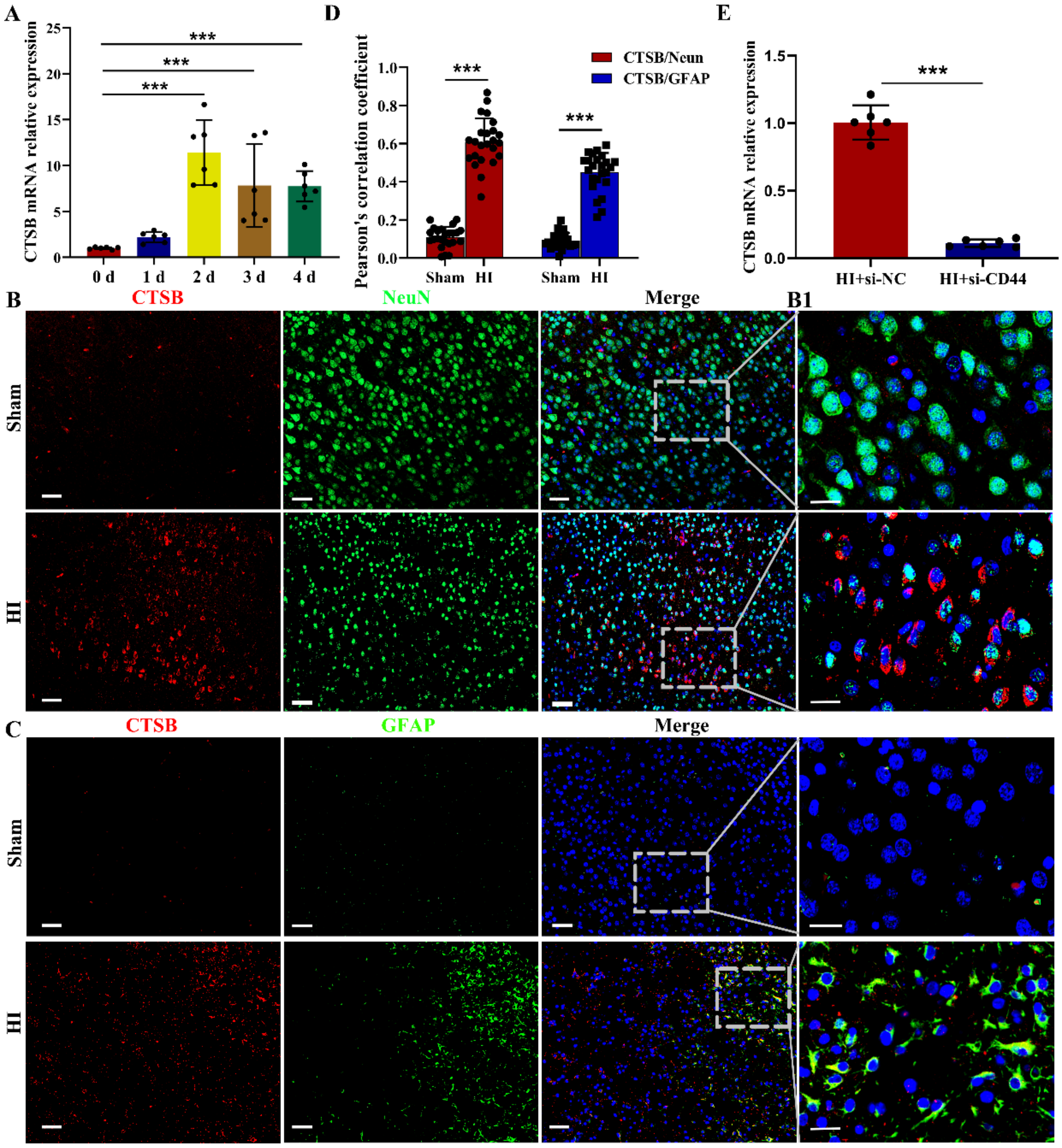

Figure S4. CTSB was localized in NeuN+ and GFAP+ cells. (**A**) Expression of CTSB mRNA in ipsilateral cortex was detected at 0 d, 1 d, 2 d, 3 d and 4 d post-HI (n=6). (**B**) CTSB was up-regulated after HI insult and located to NeuN+ neurons (n=4). Scale bar = 50 μm; B1: Magnified views of boxed regions in B showing colocalization of CTSB+ and NeuN+ cells. Scale bar = 20 μm. (**C**) CTSB was up-regulated after HI insult but less located to GFAP+ astrocytes (n=4). Scale bar = 50 μm; C1: Magnified views of boxed regions in C showing colocalization of CTSB+ and GFAP+ cells. Scale bar = 20 μm. (**D**) Visualization of the quantification of colocalization with the different markers using the Pearson's correlation coefficient. (**E**) Expression of CTSB mRNA in ipsilateral cortex following treated with si-NC or si-OPN was detected at 3 d post-HI (n=6). Values represent the mean ± SD, ***p < 0.001 according to one-way ANOVA with Dunnett corrections in (**A**). ***p < 0.001 according to *t*-test in (**D**) and (**E**). CTSB, cathepsin B; d, days; GFAP, glial fibrillary acidic protein; HI, hypoxia-ischemia; NC, negative control; NeuN, neuronal specific nuclear protein.

Figure S5

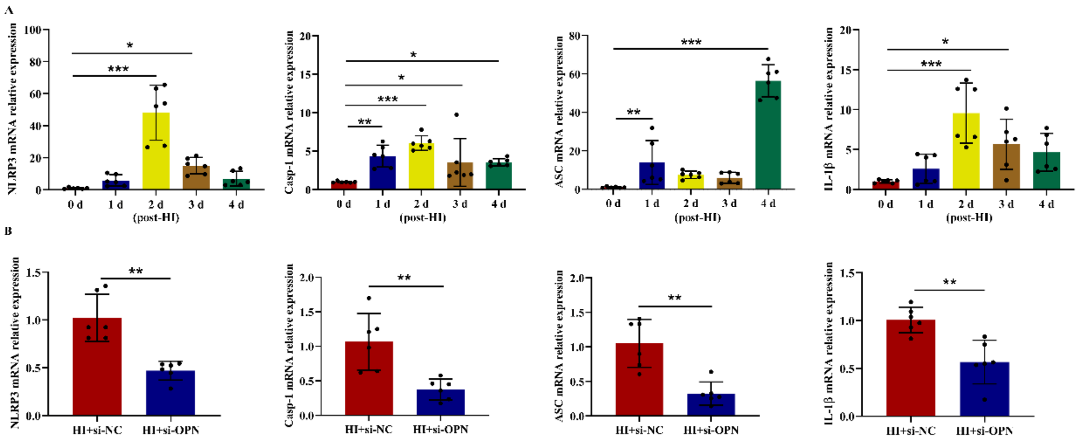

Figure S5. Change of mRNA levels of NLRP3 inflammasome. (**A**) Expression of NLRP3, Caspase-1, ASC and IL-1β mRNA in ipsilateral cortex was detected at 0 d, 1 d, 2 d, 3 d and 4 d post-HI (n=6). (**B**) Expression of NLRP3, Caspase-1, ASC and IL-1β mRNA in ipsilateral cortex following treated with si-NC or si-OPN was detected at 3 d post-HI (n=6). Values represent the mean ± SD, *$p < 0.05$, **$p < 0.01$, ***$p < 0.001$ according to one-way ANOVA with Dunnett corrections in (**A**). **$p < 0.01$ according to t-test in (**B**). ASC, apoptosis-associated speck-like protein containing a CARD; HI, hypoxia-ischemia; Casp-1, Caspase-1; d, days; IL-1β, interleukin-1β; NC, negative control; NLRP3, NOD-like receptor thermal protein domain associated protein 3; OPN, osteopontin.

Figure S6

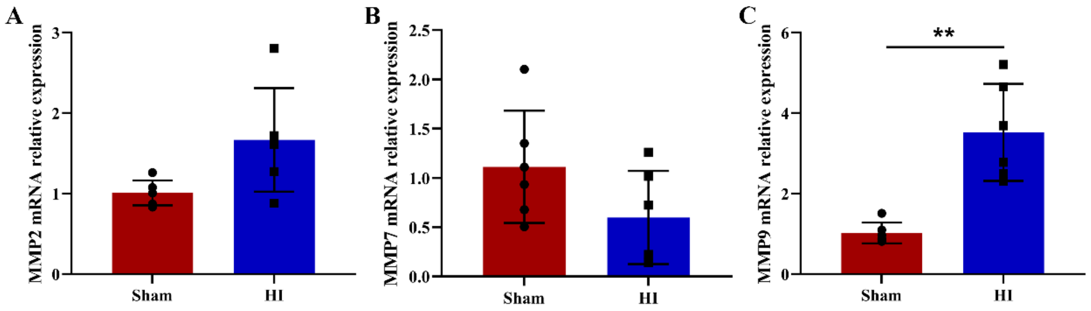

Figure S6. The MMP9 expression was observed post-HI. Expression of MMP2 (**A**) MMP7 (**B**) and MMP9 mRNA (**C**) in ipsilateral cortex was detected at 3 d post-HI by qRT-PCR (n=6). Values represent the mean ± SD, **$p < 0.01$ according to t-test. d, days; HI, hypoxia-ischemia; MMP, matrix metalloproteinases.

Figure S7

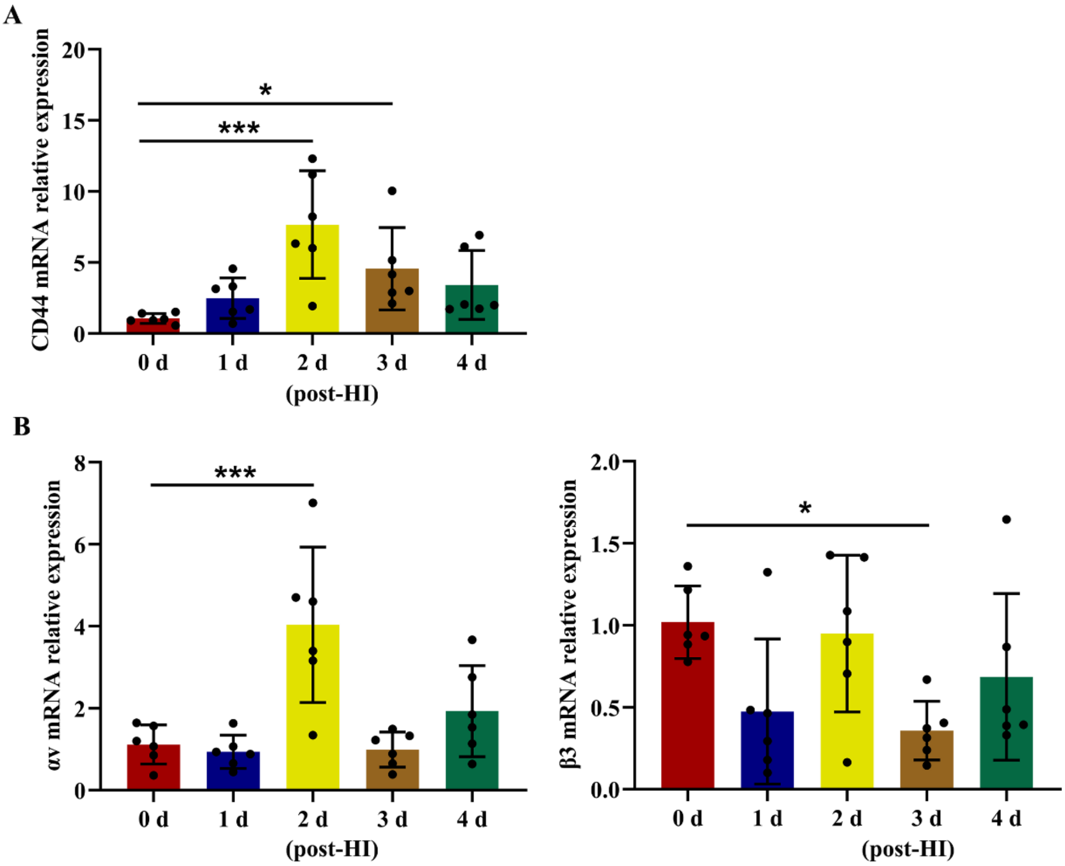

Figure S7. The CD44, αv and β3 mRNA expression was observed post-HI. Expression of CD44 (**A**), αv and β3 mRNA (**B**) in ipsilateral cortex was detected at 0 d, 1 d, 2 d, 3 d and 4 d post-HI by qRT-PCR (n=6). Values represent the mean ± SD, **p* < 0.05, ****p* < 0.001 according to one-way ANOVA with Dunnett corrections. HI, hypoxia-ischemia; d, days.

Figure S8

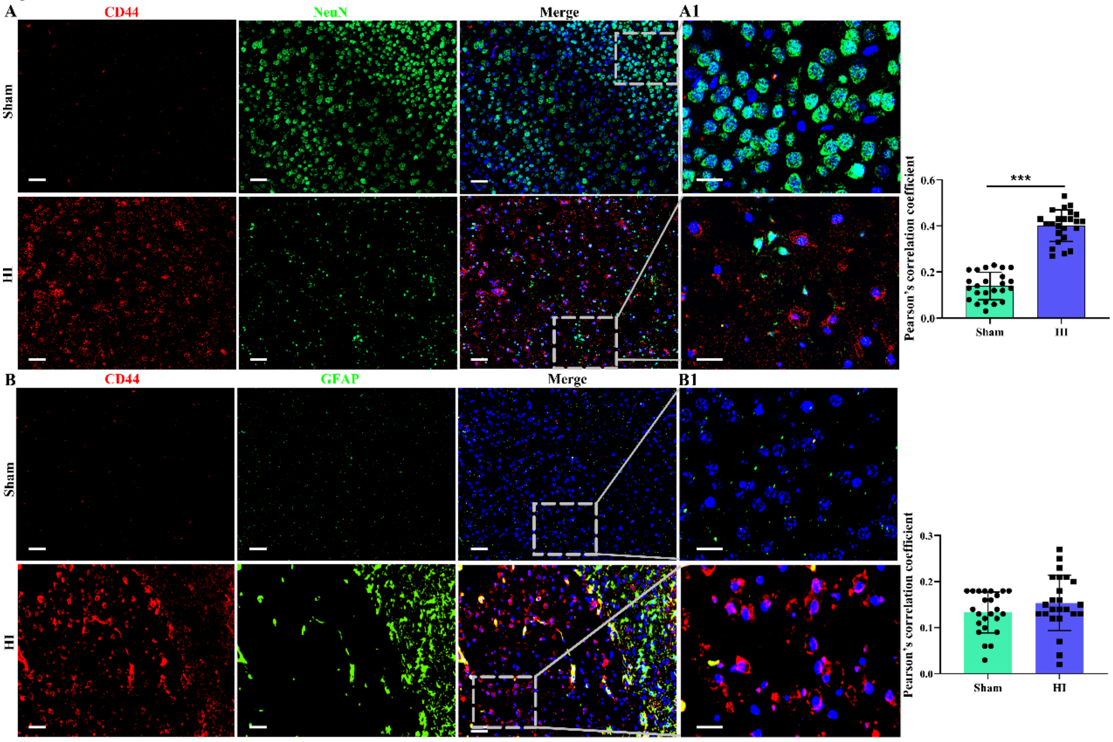

Figure S8. CD44 was colocalized in NeuN+ and GFAP+ cells. (**A**) CD44 was up-regulated after HI insult and located to NeuN+ neurons (n=4). Scale bar = 50 μm; A1: Magnified views of boxed regions in A showing colocalization of CD44+ and NeuN+ cells. Scale bar = 20 μm. (**B**) CD44 was not located to GFAP+ astrocytes (n=4). Scale bar = 50 μm; B1: Magnified views of boxed regions in B showing colocalization of CD44+ and GFAP+ cells. Scale bar = 20 μm. Values represent the mean ± SD, ***$p < 0.01$ according to *t*-test. GFAP, glial fibrillary acidic protein; HI, hypoxia-ischemia; NeuN, neuronal specific nuclear protein.

Figure S9

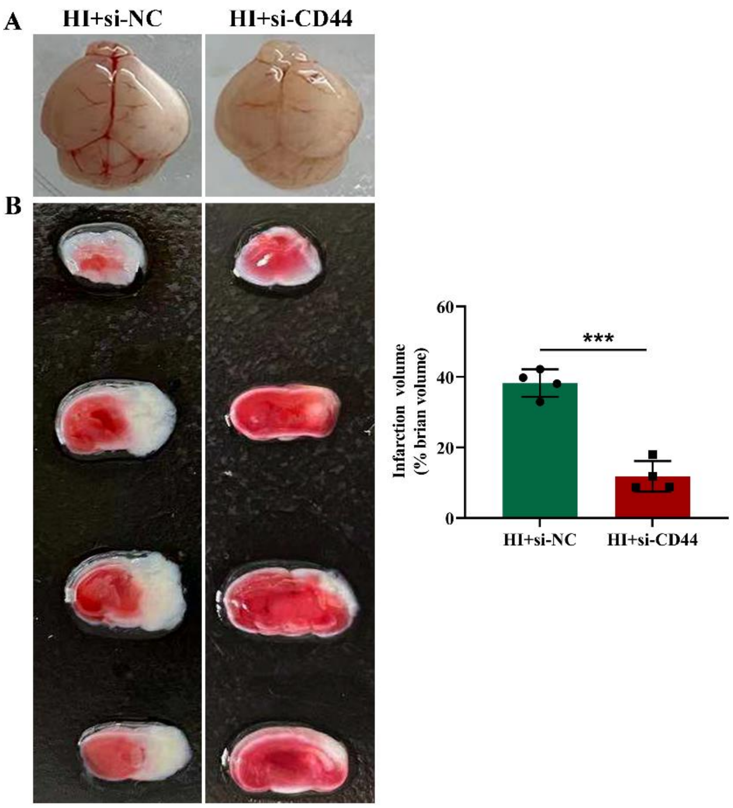

Figure S9. Silence of CD44 attenuated brain damage after HI insult. (**A**) Representative brain pictures at 3 d following HI treated with si-NC or si-CD44 (n=4). (**B**) Representative TTC staining and quantification of brain infarct volume at 3 d following HI treated with si-NC or si-CD44 (n=4). Values represent the mean ± SD, ***$p < 0.001$ according to *t*-test in (**B**). HI, hypoxia-ischemia; NC, negative control.

Figure S10

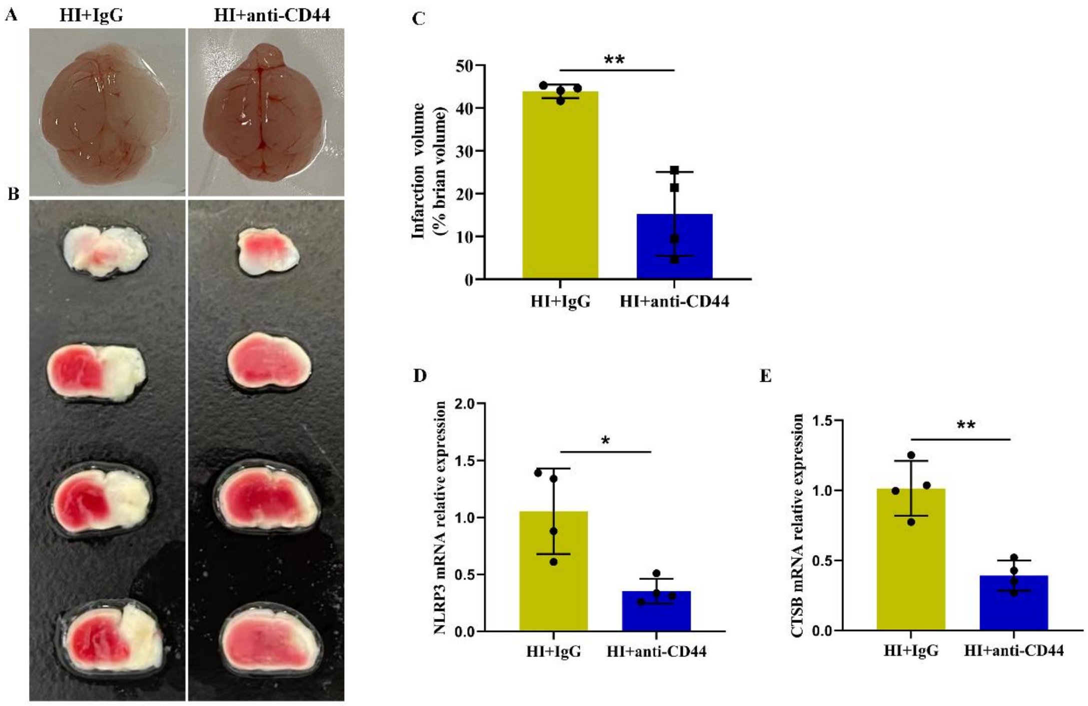

Figure S10. Anti-CD44 blocked HI induce brain damage and lysosomal. (**A**) Representative brain pictures at 3 d following HI treated with IgG or anti-CD44 (n=4). (**B**) Example of images of TTC staining at 3 d following HI treated with IgG or anti-CD44 (n=4). (**C**) Quantification of brain infarct volume. (**D**) NLRP3 and CTSB mRNA in HI**+**IgG or HI**+**anti-CD44 group were detected by qRT-PCR (n=4). Values represent the mean ± SD, *$p < 0.05$, **$p < 0.01$, according to *t*-test in (**C**-**E**). CTSB, cathepsin B; HI, hypoxia-ischemia; NLRP3, NOD-like receptor thermal protein domain associated protein 3.
